# Is Anyone Else Feeling Completely Nonessential? Meaningful Work, Identification, Job Insecurity, and Online Organizational Behavior during a Lockdown in The Netherlands

**DOI:** 10.3390/ijerph19031514

**Published:** 2022-01-28

**Authors:** Jaap W. Ouwerkerk, Jos Bartels

**Affiliations:** 1 Department of Communication Science, Vrije Universiteit Amsterdam, 1081 HV Amsterdam, The Netherlands; j.w.ouwerkerk@vu.nl; 2 Department of Communication Studies, School of Communication, Hong Kong Baptist University, Hong Kong, China

**Keywords:** COVID-19, lockdown, meaningful work, identification, job insecurity, organizational citizenship behavior

## Abstract

COVID-19 has affected employees worldwide, and in many countries, governments have used lockdowns to control the pandemic. In some countries, employees were divided into essential and nonessential workers. A survey among Dutch employees (N = 408) investigated how a lockdown in response to the pandemic affected work perceptions. The study found that employees who were not working during lockdown, or whose work hours were reduced sharply, perceived their job as contributing less to the greater good, identified less strongly with their organization, and experienced more job insecurity compared with those who retained a large percentage of their work activities. The longer employees were in lockdown, the weaker their greater-good motivations and the more job insecurity. Furthermore, identification with colleagues and perception of positive meaning in one’s job were significant predictors of online organizational citizenship behavior directed at other individuals (OCB-I), whereas organizational identification predicted such behavior directed at the organization (OCB-O). Moreover, indicative of a job preservation motive, increased job insecurity was related to more online OCB-O, and more deviant online behaviors directed at others in the form of cyberostracism and cyberincivility. We further discuss practical lessons for future lockdowns to minimize negative consequences for organizations and employees.

## 1. Introduction

When the Dutch government announced its first ”intelligent” lockdown in March 2020 in response to the COVID-19 pandemic and published a list of essential jobs, a comedian noted in a national radio commentary: “Everywhere there is applause for healthcare workers, police, postal workers, and cleaners, but I have not heard anyone (talking) about what that list also means—that millions of Dutch people have just been told that they are doing meaningless work” [1]. Similarly, Ruby Buddemeyer [2] wrote an op-ed in *Cosmopolitan* with the telling subtitle, “Is anyone else feeling completely unessential?” These examples illustrate that a government-ordered lockdown that strongly emphasizes the difference between essential and nonessential workers inadvertently may impact the extent to which people feel that their jobs have positive meaning or contribute to the greater good see [3]. A lockdown also may affect other work perceptions. Working less during a lockdown decreases contact with one’s colleagues and organization, posing a possible threat to feelings of identification with these entities e.g., [4]. Moreover, being asked to perform fewer work activities because one’s job has been deemed nonessential likely will elicit perceptions of job insecurity, considering that the existence of one’s job could be at stake [5,6].

Since the beginning of the pandemic, a vast number of studies have been conducted on the consequences of this dramatic event for employee job satisfaction [7,8], mental and physical well-being [9] and, to a lesser extent, behavior and employee well-being [10]. Moreover, some research focuses specifically on employee reactions toward a lockdown in response to COVID-19. Studies, for example, focus on the relationship between COVID-19-induced stressors on employees’ distress levels and job performance [11], working from home and perceived employee happiness [12], work loss during coronavirus lockdown and short-term health [13], and obligatory confinement due to COVID-19 and self-perceived health [14]. However, none of these studies have focused on the differences between employees with so-called governmental imposed meaningful jobs versus unmeaningful jobs. We believe that this distinction is crucial for explaining different employee responses to a lockdown. Moreover, employees’ identification with the organization, which has proven to be a powerful predictor of employee attitudes and behaviors toward the organization in a wide variety of contexts e.g., [15,16], seems to be overlooked in studies on COVID-19 lockdowns. Finally, to date, none of these previous studies have focused on the consequences of a lockdown on employees’ online positive and negative behavior towards colleagues and the organization. The current study extended previous research in several ways.

**Figure 1:**
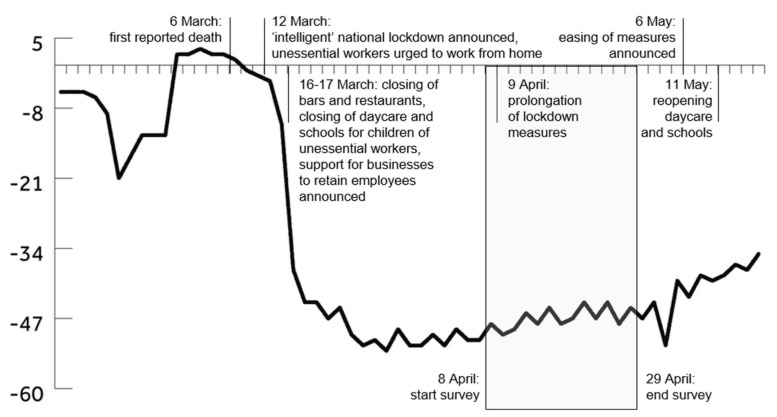
Timeline of the first lockdown in the Netherlands. The vertical axis displays the estimated percentage of decline in crowding of workplaces on workdays compared to normal according to Google Community Mobility Reports based on [24].

The present study aimed to assess the impact of not working during a lockdown or experiencing sharply reduced work hours on perceptions of meaningful work, identification, and job insecurity. Furthermore, we investigated the relationships between these work perceptions and prosocial and deviant online organizational behaviors among employees who still (partly) were working. Recent research has found that risk threats to employees can influence citizenship behavior [17]. The current study focused on online citizenship behaviors, since COVID-19 lockdowns have forced a lot of work to be performed online [12,18,19]. We surveyed Dutch workers during three lockdown weeks in April 2020 before government measures started to ease and workplaces became more crowded again (see Figure 1). We used the conservation of resources (COR) theory to analyze and explain the results. COR has been used to explain the motivation that drives humans to both maintain their current resources and pursue new ones [20,21,22,23]. Hobfoll [22] proposed this theory as a way to explain factors that cause (psychological) stress. COR asserts that stress can be the consequence of (1) sensing a threat to resources, (2) actual loss of resources, or (3) a lack of gained resources. In considering that dividing jobs into nonessential and essential as a consequence of a COVID-19 lockdown can lead to much stress among employees, we deemed COR theory appropriate for analyzing factors that influence employees’ positive and negative citizenship behaviors.

## 2. Theory and Hypotheses Development

### 2.1. Conservation of Resources

Conservation of resources (COR) theory is one of the most dominant theories for explaining human stress and well-being [21,25]. COR suggests that individuals possess only a limited supply of resources and that they want to obtain and build them while protecting them from real or potential threats [26]. Hobfoll et al. [25] stated that “resources include object resources (e.g., a car, tools for work), condition resources (e.g., employment, tenure, seniority), personal resources (e.g., key skills and personal traits, such as self-efficacy and optimism), and energy resources (e.g., credit, knowledge, money)” (p. 105). These resources’ significance differs based on people’s priorities, previous experiences, and circumstances [21]. When people retain, gain, and build resources, they feel relaxed and safe [25]. However, when individuals perceive threats to their existing resources, they can experience certain levels of stress and emotional exhaustion [27]. Consequently, this can lead to a decrease in personal well-being and even burnout [23]. Several researchers have concluded that context factors—such as job nature, organizational climate, and organizational support—act as boundaries that potentially signal to an individual how resource-draining a trigger is or how valuable certain resources are [21,28,29]. The loss of people’s psychological resources is an apparent result of the COVID-19 pandemic [30,31]. Several studies already have examined employee well-being in the COVID-19 context [32,33,34,35] as an outcome of different resources. COR-driven studies have examined, e.g., survivors’ COVID-19-related stress and performance [36], fear of COVID-19 with regard to employees’ job insecurity and emotional exhaustion [32], perceived stress and psychological well-being [37], and turnover intentions [31]. The present study investigates the consequences from loss of employee resources resulting from the first lockdown in the Netherlands from a COR perspective.

### 2.2. Relationships between Work Left during Lockdown and Work Perceptions

It is important that employees perceive their work as holding positive meaning and being significant [38]. A meta-analytic review indicated that such perceptions of meaningful work are related positively to desirable work outcomes and negatively to undesirable work outcomes [39]. In the present study, we focused on two dimensions of meaningful work identified by Steger et al. [38] that indicate the strongest correlations with work-related variables. The first, positive meaning, reflects the subjective experience that what one is doing holds personal significance. The second, greater-good motivations, is related to the concept of calling [40] and reflects the desire to impact the greater good. Kramer and Kramer [3] have suggested that these perceptions of meaningful work could be altered by changes in the status of occupations during the COVID-19 pandemic. In line with this notion, we suggest that not working or experiencing sharply reduced work hours during a lockdown may elicit or accentuate the notion among some employees that they have what has been termed by anthropologist and activist David Graeber [41] as a ”bullshit job.” Graeber already imagined that the sudden vanishing of certain jobs would make it more apparent that these were less essential for the functioning of society to begin with. However, being viewed as essential by government and society, thereby maintaining most work activities during a lockdown, may strengthen the idea that one is doing meaningful work. Based on COR assumptions, not working or experiencing reduced work hours could be perceived as a loss of resources and lead to negative feelings and thus weakening positive meaning. Therefore, we propose the following hypotheses:

**Hypothesis** **1a** **(H1a).** *Employees who are not working during a lockdown or have experienced sharply reduced work hours will perceive their jobs as having less positive meaning compared with those who still are performing a high percentage of their work activities*.

**Hypothesis** **2a** **(H2a).** *Employees who are not working during a lockdown or have experienced sharply reduced work hours will perceive their jobs as contributing less to the greater good compared with those who still are performing a high percentage of their work activities*.

Employee identification, i.e., “the perception of oneness with or belongingness to an organization” [42] (p. 104), has been recognized as a crucial factor in understanding work behavior e.g., [43]. Under normal circumstances, the basis for this perceived bond between employees and their organization relies on cues, e.g., dress codes and organizational routines; organizational identifiers, e.g., shared office buildings and co-location of employees; and frequent face-to-face communication [44]. However, working less during a lockdown implies decreased contact with one’s organization, thereby posing a possible threat to employee identification. Indeed, the degree of physical isolation among virtual employees has been linked to lower levels of organizational identification [4]. Moreover, some researchers have argued that direct face-to-face contact is even more important in fostering identification with subgroups or teams within an organization e.g., [45]. Based on these notions, we propose the following hypotheses:

**Hypothesis** **2a** **(H2a).** *Employees who are not working during a lockdown or have experienced sharply reduced work hours will identify less strongly with their organizations compared with those who still are performing a high percentage of their work activities*.

**Hypothesis** **2b** **(H2b).** *Employees who are not working during a lockdown or have experienced sharply reduced work hours will identify less strongly with their direct colleagues compared with those who still are performing a high percentage of their work activities*.

In many countries, unemployment has increased sharply during COVID-19 pandemic lockdowns, and this also was the case in the Netherlands, although the impact has been limited and delayed by the announcement, during the first week of lockdown, of financial support packages for industries and businesses with the aim of retaining employees as long as possible (see Figure 1). However, workers who were asked or forced to perform fewer work activities because their jobs were deemed nonessential arguably were likely to experience high levels of job insecurity during lockdown, i.e., they were likely to have overall concerns about the continued existence of their jobs in the future, which may lead to various short- and long-term undesirable outcomes [46]. In line with COR assumptions, we propose the following hypothesis:

**Hypothesis** **3** **(H3).** *Employees who are not working during a lockdown or have experienced sharply reduced work hours will experience more job insecurity compared with those who still are performing a high percentage of their work activities*.

### 2.3. Relationships between Work Perceptions and Online Organizational Behaviors during Lockdown

Several scholars e.g., [47,48] have argued that not only task performance is crucial for organizational effectiveness, but also organizational citizenship behavior (OCB) and counterproductive work behavior (CWB). OCB is discretionary and often not rewarded, but nonetheless improves the organization [49] and is directed at other individuals (e.g., helping a colleague) or the organization as a whole (e.g., defending the organization in public), referred to as OCB-I and OCB-O, respectively [50]. However, CWB harms an organization [51]. Such behavior also can be directed at other individuals (e.g., being rude to a colleague) or the organization as a whole (e.g., taking longer-than-necessary breaks). In the present study, we focused on online expressions of OCB-I (e.g., helping a colleague via social media) and OCB-O (e.g., responding positively to messages about one’s organization on social media) during lockdown. Moreover, we investigated online expressions of CWB directed at individuals during lockdown in the form of cyberincivility [52] that may involve either active cyberincivility (e.g., sending an angry email) or passive acts of cyberostracism (e.g., ignoring an email).

Steger et al. [38] demonstrated that viewing one’s job as having positive meaning and contributing to the greater good are both positively related to OCB. Moreover, Allen et al. [39] observed small-to-moderate positive meta-analytic relationships between perceptions of meaningful work and OCB. Extant research directly investigating the relationship between meaningful work and CWB is limited, but a study by Usman et al. [53] found that the dimensions of meaningful work as identified by Steger et al. [38] are related to a reduction in online deviant organizational behavior in the form of cyberloafing. Moreover, following COR, meaningful work could be viewed as an important outcome for employees [54]. Therefore, we propose the following hypotheses:

**Hypothesis** **4a** **(H4a).** *The more employees view their jobs as having positive meaning, the more they engage in online organizational citizenship behavior (OCB-I and OCB-O) during lockdown*.

**Hypothesis** **4b** **(H4b).** *The more employees view their jobs as contributing to the greater good, the more they engage in online organizational citizenship behavior (OCB-I and OCB-O) during lockdown*.

**Hypothesis** **4c** **(H4c).** *The more employees view their jobs as having positive meaning, the less they engage in cyberostracism and cyberincivility during lockdown*.

**Hypothesis** **4d** **(H4d).** *The more employees view their jobs as contributing to the greater good, the less they engage in cyberostracism and cyberincivility during lockdown*.

Organizational identification is an important predictor of motivation at work, as strongly identified employees are more likely to engage in behaviors that benefit their work group or organization e.g., [55,56]. Accordingly, meta-analytic reviews indicate a robust empirical link between organizational identification and OCB [43,57,58]. However, when considering the relationship between identification and OCB, congruence between the focus of identification and the OCB dimension under investigation is important [16], i.e., extant research indicates that identifying with coworkers or one’s department increases OCB-I, while identification with the organization increases OCB-O e.g., [59]. Therefore, we propose the following hypotheses:

**Hypothesis** **5a** **(H5a).** *The more strongly employees identify with their direct colleagues, the more they engage in online organizational citizenship behavior directed at other individuals (OCB-I) during lockdown*.

**Hypothesis** **5b** **(H5b).** *The more strongly employees identify with their organization, the more they engage in online organizational citizenship behavior directed at the organization (OCB-O) during lockdown*.

Considering that extant research has focused predominantly on the relation of identification with positive work behavior, evidence of a link between identification and deviant organizational behavior is relatively scarce. However, some recent studies have demonstrated a negative relationship between organizational identification and CWB e.g., [60,61]. Moreover, a meta-analysis indicates that a strongly related concept, organizational commitment, demonstrates a reliable average negative correlation with CWB [62]. In line with COR assumptions, identification also can be a buffer resource to prevent negative employee behavior. As noted earlier, the specific focus of identification (toward individuals or the organization as a whole) is important when considering the relationship with work behavior. As cyberostracism and cyberincivility comprise online expressions of CWB directed at other individuals, we propose the following hypothesis:

**Hypothesis** **5c** **(H5c).** *The more strongly employees identify with their direct colleagues, the less they engage in cyberostracism and cyberincivility during lockdown*.

Empirical evidence linking job insecurity to OCB is equivocal [63,64], with some studies reporting a negative relationship e.g., [65] and others demonstrating a positive one e.g., [66] or indicating no relationship e.g., [67]. Similarly, studies linking job insecurity to CWB report both negative correlations e.g., [68] and positive correlations e.g., [65]. Indeed, Shoss [64] identified different psychological mechanisms to explain the link between job insecurity and outcome measures, leading to corresponding and competing predictions regarding workplace behavior.

First, stress-related mechanisms suggest that job insecurity may result in displaced aggression and diminished self-regulation, thereby increasing interpersonal mistreatment and other forms of CWB. Second, social exchange-related mechanisms suggest that job insecurity reflects a breach of the relational psychological contract, resulting in less OCB and more CWB to restore balance. As a third mechanism, Shoss [64] identified a job preservation perspective that suggests job insecurity should motivate workers to demonstrate their worth to their employer by devoting extra effort toward behaviors that will be noticed and valued, implying increased OCB directed at the organization as a whole. At the same time, a job preservation motive predicts that employees might mistreat coworkers as a means of competing with potential rivals [69]. From this perspective, job insecurity may increase OCB-O and CWB-I simultaneously. However, given the conflicting predictions, we did not formulate specific hypotheses concerning the relationship between job insecurity and online organizational behaviors during lockdown and instead present the following research question:

RQ: How does job insecurity affect online organizational behavior during lockdown in the form of OCB-I, OCB-O, cyberostracism, and cyberincivility?

## 3. Materials and Methods

### 3.1. Participants and Procedure

The present study was conducted online using internet-based survey software. The survey was distributed via a snowball procedure that entailed distributing a link in emails and social media messages directed at contacts within the researchers’ networks and a request to forward this link. Before providing consent and checking whether the requirement of having a paid job was met, respondents were told that the research concerned how people have experienced work since the COVID-19 pandemic began, emphasizing that, in accordance with ethical standards in scientific research, their answers would be kept private, and their identities would be anonymized. Altogether, 545 respondents were surveyed during a three-week lockdown period in April 2020 (8–29 April; see Figure 1). Of this sample, 542 provided consent, and 478 met the requirement of having a paying job. Of these 478, 408 completed the survey and were included in the analyses. This final sample comprised 160 males and 248 females (M_age_ = 29.83; SD = 12.57) from a wide variety of economic sectors (see Table 1). For more details on the sample’s composition, see the description of the background questions. The survey started with background questions about the respondent’s job, the organization they worked for, their demographics and living situation, and how work had changed since the lockdown began. This was followed by statements assessing work perceptions and questions measuring online organizational behaviors during lockdown.

### 3.2. Measures

#### 3.2.1. Control Variables

(1) Job background questions. Respondents were asked what type of job they had (full-time, *n* = 179; part-time, *n* = 107; side job combined with education, *n* = 81; paid traineeship, *n* = 7; paid internship, *n* = 34), how long they had been working for their current employer (less than six months, *n* = 63; 6–12 months, *n* = 61; 1–5 years, *n* = 152; 6–10 years, *n* = 34; 11–15 years, *n* = 25; 16–20 years, *n* = 12; or more than 20 years, *n* = 61), how many hours a week they worked before the lockdown (normal job hours; M = 29.83; SD = 12.57), and whether they had a supervisory function (*n* = 101) or not (*n* = 307).
(2) Organizational background questions. Respondents were asked to estimate the size of the organization or organizational branch they worked for (1–5, *n* = 23; 6–10, *n* = 25; 11–99, *n* = 131; 100–200, *n* = 57; 201–1000, *n* = 70; more than 1000 people, *n* = 102) and their employer’s economic sector (see Table 1).
(3) Personal background questions. Respondents also were asked for their gender at birth (*n*_male_ = 160; *n*_female_ = 248), age (M = 29.83; SD = 12.57), and highest completed education level (no education, *n* = 0; primary school, *n* = 0; high school, *n* = 54; lower professional education, *n* = 2; intermediate professional education, *n* = 59; higher professional education, *n* = 157; university, *n* = 136). Furthermore, respondents were asked whether they lived with others (a partner, parents, or in a communal living arrangement, *n* = 337) or alone (*n* = 71). We also asked whether their household included any children (*n*_yes_ = 116); if so, whether these children required care during the day (*n*_yes_ = 40); and if so, whether they had to provide childcare themselves during lockdown (*n*_yes_ = 38). We referred to the latter measure as increased care responsibilities.
(4) Days in lockdown. Days in lockdown were assessed as the days in April 2020 (8–29 April) when the respondent completed the survey (M = 11.17; SD = 4.85).

#### 3.2.2. Work Percentage Left during Lockdown

Respondents were asked whether they were still (partly) working during lockdown (*n* = 357) or whether their work was halted completely (*n* = 51). If they were still (partly) working, they were asked to indicate on a scale ranging from zero to 100% how much of their work activities remained active during lockdown (M = 68.43%; SD = 36.46). If respondents indicated that their work was halted completely, they were scored a zero for this measure. Note that the percentage of work that remained active during lockdown corresponds with the decline in crowding of workplaces on workdays compared with normal periods based on Google Community Mobility Reports (see Figure 1) and differs significantly depending on economic sector (F(14, 393) = 14.17, *p* < 0.001, η_p_^2^ = 0.34) As can be seen in Table 1, four sectors were hit particularly hard, with less than 50% of work remaining active during lockdown: hospitality and tourism; airlines; transportation and logistics; and culture, sports, and leisure.

#### 3.2.3. (Increased) Telework Percentage

Telework (increase) was assessed on two scales ranging from zero to 100% by asking respondents who were still (partly) working how much of their work they performed at home before lockdown (M = 20.21%; SD = 27.53) and during lockdown (M = 66.76%; SD = 39.40). The difference score (during vs. before) was used as a measure of increased telework (M = 46.55%; SD = 40.53).

#### 3.2.4. Work Perceptions

Measures consisted of a statement to which participants responded on seven-point scales in terms of their own degree of agreement (1 = total disagreement, 7 = total agreement). We used the two subscales of the Work and Meaning Inventory [38] to assess positive meaning (four items, e.g., “I have found a meaningful career”; M = 5.20, SD = 1.41, α = 0.89) and greater-good motivations (three items, e.g., “The work I do serves a greater purpose”; M = 4.87, SD = 1.64, α = 0.92). We presented the solidarity subscale of the Group-Level Self-Investment Scale for Identification [70] to measure organizational identification (three items, e.g., “I feel committed to my organization”; M = 5.41, SD = 1.56, α = 0.94). The same scale was used to assess identification with colleagues (three items, e.g., “I feel committed to my colleagues”; M = 5.19, SD = 1.44, α = 0.93). Furthermore, we formulated one item to measure job insecurity as a result of the lockdown (“I am worried about keeping my job since the lockdown”; M = 3.11, SD = 2.07).

#### 3.2.5. Online Organizational Behaviors

When respondents were still (partly) working and indicated that they had remained in online contact with others in their organization by e-mail, chat, videoconferencing, or social media (*n*_yes_ = 318), 16 questions were presented to assess the frequency with which they engaged in different online organizational behaviors during lockdown using seven-point scales (1 = never, 7 = very often). Partly based on existing scales that distinguish between offline organizational citizenship behavior (OCB) directed toward other individuals (OCB-I) and toward the organization (OCB-O), we formulated four questions intended to measure online OCB-I and four questions to measure online OCB-O. Furthermore, partly based on existing scales for active and passive cyberincivility [52], we formulated four questions intended to measure cyberincivility and four questions to measure cyberostracism. An exploratory factor analysis of the 16 items was conducted to identify these online organizational behaviors’ underlying dimensions (see Appendix A, Table A1). Based on this analysis, scales were constructed to measure online OCB-I (*M* = 4.64, *SD* = 1.40, *α* = 0.80), online OCB-O (*M* = 3.60, *SD* = 1.49, *α* = 0.73), cyberincivility (*M* = 1.25, *SD* = 0.51, *α* = 0.80), and cyberostracism (*M* = 1.53, *SD* = 0.76, *α* = 0.75).

## 4. Results

### 4.1. Relationships between Work Remaining Active during Lockdown and Work Perceptions

We first tested Hypotheses 1–3 by examining the differences between employees who were not working at all during lockdown and those who were still (partly) working (see Table 2). As predicted, one-way ANOVAs indicated that employees who were not working during lockdown perceived less positive meaning in their work (H1a), had weaker greater-good motivations (H1b), identified less strongly with their organization (H2a), identified less strongly with their colleagues (H2b), and experienced more job insecurity (H3) compared with those who were still (partly) working, thereby providing initial support for our hypotheses. For a more fine-grained test of Hypotheses 1–3, we investigated the relationship between work percentage left during lockdown and work perceptions. The zero-order correlations between variables are depicted in Table 3. In line with predictions, work percentage left during lockdown had a positive correlation with greater-good motivations, organizational identification, and identification with colleagues, whereas it had a negative correlation with job insecurity. However, no significant correlation was found with positive meaning. To provide a more comprehensive test of these relationships, we conducted multiple regressions with work percentage left as a predictor, while controlling for age, gender (1 = male, 2 = female), living situation (1 = alone, 2 = other), increased care responsibilities (1 = yes, 2 = no), supervisory function (1 = yes, 2 = no), days in lockdown, and normal job hours. Note that this model significantly predicted all five work perceptions (*p*’s < 0.001; see Table 4, Table 5 and Table 6).

In line with the zero-order correlation, and unlike H1a, the multiple regression analysis predicting positive meaning did not indicate a relationship with work percentage left (see Table 4, left). Additional findings revealed that having a supervisory function and more normal (i.e., pre-lockdown) work hours were related to perceiving more positive meaning in one’s job. The multiple regression analysis predicting greater-good motivations did indicate a relationship with work percentage left (see Table 4, right). As expected, employees with sharply reduced work hours during lockdown perceived their jobs as contributing less to the greater good compared with those who still were performing a higher percentage of their work activities (H1b). Interestingly, days in lockdown also predicted greater-good motivations. That is, the longer employees were in lockdown, the weaker their greater-good motivations. Moreover, those living with others reported stronger greater-good motivations than those living alone, suggesting that having direct support at home may strengthen the notion that one’s job contributes to the greater good.

The multiple regression analysis predicting organizational identification indicated a relationship with work percentage left (see Table 5, left). As expected, employees with sharply reduced work hours during lockdown identified less strongly with their organization compared with those performing a higher percentage of their work activities (H2a). Furthermore, older employees and those with a supervisory function reported stronger identification with their organizations. Despite a significant zero-order correlation, the multiple regression analysis predicting identification with colleagues indicated no relationship with work percentage left (see Table 5, right). Thus, our more fine-grained test did not support H2b, thereby suggesting that whereas organizational identification suffers from a low percentage of work left during lockdown, identification with colleagues does not. Furthermore, as was the case for organizational identification, older employees and those with a supervisory function reported stronger identification with colleagues. Moreover, female respondents identified more strongly with colleagues.

The multiple regression analysis predicting job insecurity indicated a relationship with work percentage left (see Table 6). As expected, employees with sharply reduced work hours during lockdown experienced more job insecurity compared with those who were still performing a higher percentage of their work activities (H3). Furthermore, days in lockdown predicted job insecurity, i.e., the longer employees were in lockdown, the more job insecurity they experienced.

### 4.2. Relationships between Work Perceptions and Online Organizational Behaviors during Lockdown

To test Hypotheses 4 and 5, we conducted multiple regressions with work perceptions (positive meaning, greater-good motivations, organizational identification, identification with colleagues, job insecurity) as predictors of online organizational behaviors during lockdown, while controlling for age, gender (1 = male, 2 = female), living situation (1 = alone, 2 = other), increased care responsibilities (1 = yes, 2 = no), supervisory function (1 = yes, 2 = no), days in lockdown, normal job hours, work percentage remaining active, normal telework percentage, and increased telework percentage. Note that this model significantly predicted all four online organizational behaviors (*p*’s < 0.001; see Table 7 and Table 8).

The multiple regression analysis predicting online OCB-I indicated a relationship with both positive meaning and identification with colleagues (see Table 7, left). As expected, the more positive meaning employees perceived in their job (H4a), and the more strongly they identified with their colleagues (H5a), the more they engaged in online OCB-I. However, contrary to expectations, no relationship was found with greater-good motivations (H4b). The findings further indicated that normal (i.e., pre-lockdown) telework percentage and the increase in telework percentage during lockdown were related positively to online OCB-I, suggesting that more opportunities to engage in online OCB-I increase such behavior. Moreover, employees with a supervisory function reported more online OCB-I. The multiple regression analysis predicting online OCB-O indicated a relationship with organizational identification (see Table 7, right). As expected, the more strongly employees identified with their organization, the more they engaged in online OCB-O (H5b). However, contrary to expectations, and despite significant zero-order correlations (see Table 3), no relationship was found with positive meaning (H4a) and greater-good motivations (H4b). Relevant to our research question (RQ), increased job insecurity was related to engaging in more OCB-O. The findings further demonstrated that younger employees reported more online OCB-O, which may indicate their stronger online presence.

Contrary to our expectations, the multiple regression analysis predicting cyberostracism (see Table 8, left) indicated no relationship with positive meaning (H4c) and greater-good motivations (H4d). Moreover, despite a significant zero-order correlation (see Table 3), no relationship was found with identification with colleagues (H5c). However, relevant to our research question (RQ), increased job insecurity was related to engaging in more cyberostracism. The findings further demonstrated that younger employees reported more cyberostracism, as was the case with online OCB-O. Furthermore, as was the case with online OCB-I, employees with a higher normal (i.e., pre-lockdown) telework percentage reported more acts of cyberostracism. Employees living with others also demonstrated more cyberostracism. The multiple regression analysis predicting cyberincivility (see Table 8, right) also found no relationship with positive meaning (H4c), greater-good motivations (H4d), and identification with colleagues (H5c). However, relevant to our research question (RQ), increased job insecurity was related to engaging in more cyberincivility. The findings further indicated that employees with a supervisory function are more likely to engage in cyberincivility, as was the case with OCB-I.

## 5. Discussion

### 5.1. Implications

Kramer and Kramer [3] suggested that changes in occupational status during the COVID-19 pandemic could alter perceptions of meaningful work. In line with this notion, the present study indicated that employees who were not working at all during a lockdown in the Netherlands perceived their jobs as having less positive meaning and contributing less to the greater good compared with those who were still (partly) working. Furthermore, as predicted, employees not working at all during lockdown reported less identification with their colleagues and organization, thereby corroborating research on physical isolation’s detrimental effects [4] and underlining the importance of direct contact and the presence of organizational identifiers, such as the colocation of employees, as suggested by Wiesenfeld et al. [44]. Moreover, our results demonstrate that not working during lockdown, or having reduced work hours, is linked strongly to feelings of job insecurity. In line with COR, the present study indicates that occupational status during the COVID-19 pandemic can be an important resource for employees, and that losing this resource can lead to negative consequences [22].

However, it should be noted that although we found a difference between employees who were not working at all and those still (partly) working with respect to perceiving positive meaning in one’s job and identification with colleagues, a more fine-grained test of our hypotheses revealed that the percentage of work left during lockdown did not predict these work perceptions. This suggests that it is not so much the percentage of work remaining active during lockdown that fosters positive meaning and identification with colleagues, but rather the fact that one has any work activities remaining at all. In other words, our findings indicate that having only a small amount of work during lockdown could be a sufficient resource with which to maintain perceptions of positive meaning and feelings of identification with colleagues. A first social implication of this finding is that this implies that organizations, even when normal work activities have halted completely during lockdown—as is often the case, e.g., in the hospitality industry—may want to ensure some minimal level of work activities for their employees. This could be achieved relatively easily by creating new work activities (e.g., making or testing plans for reopening after lockdown). Creating these new work activities, could then possibly reduce feelings of inequality by “nonessential” employees compared to their “essential” counterparts.

Arguably, the detrimental effects from lockdown on other work perceptions may be more difficult to counteract. Indeed, our findings demonstrate that the differences between employees not working at all and those still (partly) working were most pronounced with respect to job insecurity and greater-good motivations. To make matters worse, the present study suggests that a lockdown’s duration may contribute to these detrimental effects, i.e., the results demonstrated that employees who completed our survey at a later date during the three-week lockdown period reported more job insecurity and weaker greater-good motivations. However, this does not mean that governments and organizations cannot do anything to minimize a lockdown’s negative effects on these perceptions of work. Governments can offer financial support packages for industries and businesses to help retain employees as long as possible, reducing possible poverty issues for “nonessential” employees and preventing an economic decline.

Extant research has found that although overall welfare generosity levels in countries do not elicit systematic effects on whether or not employees feel secure, social protection measures can reduce feelings of job insecurity [71]. Governments also should consider how they frame differences between jobs when announcing a lockdown to avoid unintended negative effects on greater-good motivations. For example, they could emphasize that so-called “nonessential” jobs are vital for economic recovery after a lockdown. Still having an ”nonessential” job does provide employees with more resources than when they lose their job.

Considering that organizational support is one possible contextual resource [25], organizations also can play an important role in reducing feelings of job insecurity during lockdown by keeping employees informed at all times. For example, a study by Mauno and Kinnunen [72] revealed that inadequate organizational communication is one of the most important predictors of job insecurity. Indeed, a meta-analytic review [73] indicated a moderately negative relationship between perceptions of organizational communication and feelings of job insecurity.

Organizational communication also may play a crucial role in maintaining a sense of identification with one’s organization as a whole during lockdown. Interestingly, extant research suggests that electronic communication, rather than direct face-to-face communication, is more important for fostering identification with one’s organization. A study by Millward et al. [45] indicated that, whereas face-to-face communication was related more strongly to identification with one’s department than with one’s organization, the reverse was true for electronic communication (i.e., email). Indeed, Reicher et al. [74] have suggested that under certain conditions, face-to-face contact actually could hamper people’s sense of identification, while computer-mediated communication could strengthen it. They argued that computer-mediated communication can direct attention to the shared cognitive representation of one’s group (i.e., the organization), rather than specific interpersonal relationships within this group. This suggests that when employees are isolated physically during lockdown, organizations can maintain (or even strengthen) employee identification through computer-mediated communication. An organization’s high quality of computer-mediated communication can reduce feelings of a loss of resources as a consequence of physical isolation, possibly preventing employee burnout.

The importance of counteracting the negative consequences from not working or from reduced work hours on various work perceptions is highlighted by the present study’s finding that these perceptions are related to online organizational behaviors during lockdown. As expected, identification with colleagues emerged as a significant predictor of online organizational citizenship behavior directed at other individuals, whereas organizational identification predicted such online behavior directed at the organization, thereby corroborating previous research e.g., [59] in an online context and underlining the importance of distinguishing between different identification foci when investigating relationships with work behavior [16]. Furthermore, we found evidence of a predicted negative relationship between identification with colleagues and deviant online behavior in the form of cyberostracism. However, it should be noted that this link could be confirmed only on the basis of a zero-order correlation. Moreover, perceiving positive meaning in one’s job emerged as a predictor of online organizational citizenship behavior directed at other individuals, although other expected relationships between perceptions of meaningful work and online organization behavior were not observed when controlling for other variables, thereby suggesting that perceiving one’s work as having positive meaning and contributing to the greater good exert a relatively minor impact on online behavior during a lockdown.

However, job insecurity emerged as a significant predictor of online organizational citizenship behavior directed at the organization, as well as deviant online behaviors directed at other individuals in the form of cyberostracism and cyberincivility. Thus, job insecurity was linked simultaneously with more online expressions of OCB-O and CWB-I. This finding is not only important for understanding online organizational behaviors during lockdown, but also for our knowledge about the consequences of job insecurity during a crisis in general, as most studies examining job insecurity do so during periods of relative stability cf. [75]. However, the present study’s findings suggest that during a crisis, job preservation motivation prevails cf. [64], encouraging workers to demonstrate their worth to their employers through increased online OCB directed at the organization (e.g., defending the organization via email, chat, videoconferencing, or social media), as well as to compete with potential rivals by mistreating colleagues (e.g., using all capitals in an email to ”shout”) or even sabotaging coworkers (e.g., not forwarding an email that is important to them). Thus, especially during a crises like the COVID-19 pandemic, organizations must stay alert to possible threats to equal opportunities for the “nonessentials” versus the “essentials”.

### 5.2. Limitations and Future Research

When a national lockdown in the Netherlands began in March 2020, we were interested in how not working or working less as a result of this drastic measure would affect work perceptions. Thus, we immediately started to construct a survey and quickly distributed it via a snowball procedure. As we had no way of knowing how long the lockdown would last, and this opportunity presented itself, time was of the essence. Obviously, taking more time to organize the survey’s distribution, e.g., through a commercial panel, could have elicited a more representative sample of Dutch workers. However, as noted, we still were able to distribute the survey among workers across a wide spectrum of economic sectors affected differently with respect to the percentage of work activities left during lockdown. Moreover, we controlled for various personal and work-related factors in our analyses.

Spector [76] noted that at this point in a research article, the authors usually acknowledge the inferiority of cross-sectional design for identifying causal relationships and recommend using a longitudinal design for future research. However, according to Spector [76], “the ability of the longitudinal design to reflect causality has been overstated, and it offers limited advantages over the cross-sectional design in most cases in which it is used” (p. 125). Although we agree with this observation’s sentiment, we also acknowledge the important benefits of longitudinal research when investigating a lockdown’s long-term consequences on work perceptions and behavior. Whereas its ability to reflect causality may be overstated, it also enables researchers to capture changes over time in a turbulent situation. Indeed, time-related factors could be important when investigating a lockdown’s effects, as illustrated by our own findings that the lockdown’s duration influenced respondents’ perceptions of greater-good motivations and job insecurity. Moreover, the relevance of the empirical basis for making predictions may change rapidly during a crisis, such as the current pandemic. For example, we predicted that experiencing fewer work activities remaining active during lockdown (i.e., less direct contact with other employees) would lower employee identification, partly based on a study by Bartel et al. [4] that demonstrated that virtual workers’ degree of physical isolation decreases organizational identification. However, this study found that this effect is mediated by the lack of perceived respect that virtual workers generally experience. Given that working from home may become ”the new normal” and more widely accepted quickly, the detrimental effects on perceived respect as experienced by current virtual workers may weaken, thereby possibly also changing the link between physical isolation and identification cf. [77].

Another limitation in the present study is its reliance on single-source self-reported data. Using alternative data sources such as peer and supervisor reports in future research could serve as a control for sources of method variance e.g., [78]. However, it should be noted that the work perceptions under investigation in the present study were difficult to assess by others, even under normal circumstances. The same was true for frequency with which employees engaged in various online prosocial and deviant behaviors. Indeed, others have argued that although some OCBs and CWBs may be observable by others, most of these behaviors may be difficult to notice or are intentionally hidden e.g., [62,79]. Consequently, self-reported OCB and CWB measures show better discriminant validity compared with ratings by others, which often reflect general impressions, considering that a more objective basis for making judgments is lacking e.g., [62,76,79].

## 6. Conclusions

Many countries have taken drastic measures to contain the COVID-19 pandemic, with governments implementing various forms of local or national lockdowns, often urging or forcing people in ”nonessential” jobs to work from home as much as possible or to stop working altogether. Policy makers often are well-informed about such measures’ public health benefits and economic costs, but must make decisions with only limited information about the implications for individual employees’ well-being. The present study demonstrates that not working, or working less, as a consequence of a government-ordered lockdown is related to employees perceiving their jobs as having less meaning, identifying less strongly with their colleagues and organization, and experiencing more job insecurity. Although the pandemic has captured scientists’ attention significantly, with about 4% of all scientific output from February to July 2020 focusing on this topic, the vast majority of these papers examined medical and biological issues, and 60% were opinion pieces, not reports containing original data [80]. Therefore, we urge researchers in the organizational psychology domain to gather empirical data that can help governments and other organizations better understand lockdowns or other COVID-19 measures’ impact on individual employees. In doing so, they not only would make a vital and urgently needed scientific contribution, but also may feel slightly less nonessential.

## Figures and Tables

**Table 1:** Distribution of respondents among sectors and differences in work percentage left between sectors.

Sector:	*N*	s	Work % Left	
			M	SD
Hospitality and Tourism	35	8.6	24.54 _a_	39.08
Airline Industry	21	5.1	26.29 _a_	33.60
Transport and Logistics	11	2.7	40.36 _ab_	38.91
Culture, Sports, and Leisure	13	3.2	45.08 _ab_	41.02
Commercial Services	29	7.1	58.69 _bc_	37.21
(Retail) Trade	46	11.3	70.15 _bcd_	34.84
Government and Public Administration	33	8.1	77.1 2_cd_	28.76
Education and Science	29	7.1	78.62 _cd_	27.90
Healthcare and Welfare	59	14.5	79.10 _cd_	26.14
Media and Communication	12	6.6	85.22 _cd_	17.18
Justice and Security	16	3.9	90.63 _d_	15.63
Financial Services	26	6.4	90.77 _d_	18.72
Information Technology	13	3.2	93.00 _d_	9.37
Manufacturing, Production, and Construction	15	3.7	93.60 _d_	11.72
Other	35	8.6	66.66 _bcd_	36.49

Note: N = 408; Means with different subscripts in a column (M) are different at the 0.05 significance level using Tukey’s HSD.

**Table 2:** Differences between employees who were not working during lockdown and those who were still (partly) working.

	Not Working (*n* = 51)		Still (Partly) Working (*n* = 357)				
Variable:	*M (SD)*	95% CI	*M (SD)*	95% CI	*F* (1406)	*p*	η_p_^2^
Positive Meaning	4.75 (1.48)	[4.33, 5.16]	5.27 (1.39)	[5.12, 5.41]	6.22	0.013	.015
Greater-Good Motivations	4.22 (1.61)	[3.77, 4.67]	4.96 (1.63)	[4.80, 5.13]	9.29	0.002	.022
Identification with Organization	4.93 (1.77)	[4.44, 5.43]	5.48 (1.52)	[5.32, 5.64]	5.54	0.019	.013
Identification with Colleagues	4.81 (1.62)	[4.35, 5.27]	5.25 (1.41)	[5.10, 5.39]	4.12	0.043	.010
Job Insecurity	4.37 (2.00)	[3.81, 4.93]	2.93 (2.02)	[2.72, 3.14]	22.79	0.000	.053

**Table 3:** Correlations between variables.

	1	2	3	4	5	6	7	8	9	10	11	12	13
1. Normal Job Hours													
2. Work % Left	0.33 ***	--											
3. Normal Telework %	0.12 *	0.03	--										
4. Increase Telework %	0.14 **	0.22 ***	–0.38 ***	--									
5. Positive Meaning	0.22 ***	0.08	–0.06	–0.02	--								
6. Greater-Good Motivations	0.13 **	0.16 **	–0.10	0.02	0.51 ***	--							
7. Identification with Organization	0.16 **	0.17 **	–0.09	0.07	0.57 ***	0.33 ***	--						
8. Identification with Colleagues	0.19 ***	0.14 **	–0.04	-0.00	0.51 ***	0.31 ***	0.76 ***	--					
9. Job Insecurity	–0.04	–0.30 ***	–0.05	0.03	0.05	–0.09	–0.04	.01	--				
10. Online OCB-I	0.23 ***	0.19 ***	0.10	0.20 **	0.24 ***	0.06	0.25 ***	0.31 ***	0.08	--			
11. Online OCB-O	0.09	–0.02	–0.01	–0.00	0.29 ***	0.13 *	0.29 ***	0.2 6 ***	0.18 **	0.40 ***	--		
12. Cyberostracism	0.07	–0.01	0.14 **	–0.04	–0.10	–0.09	–0.18 ***	–0.14 *	0.12 *	0.09	0.22 ***	--	
13. Cyberincivility	0.11 *	–0.07	0.10	–0.08	–0.09	–0.05	–0.15 **	–0.09	0.10	0.03	0.14 *	0.43 ***	--

Note: *N* = 408 with exception of telework (*n*_3–4_ = 357) and online organizational behaviors (*n*_10–13_ = 318); * *p* < 0.05; ** *p* < 0.01; *** *p* < 0.00.

**Table 4:** Multiple regression analysis of work percentage left on positive meaning and greater-good motivations.

	Positive Meaning				Greater-Good Motivations	
	*B (SE B)*	95% CI	*p*	*B (SE B)*	95% CI	*p*
Age	0.01 (0.01)	[–0.00, 0.02]	0.117	0.00 (0.01)	[–0.01, 0.02]	0.545
Gender	0.26 (0.15)	[–0.03, 0.55]	0.080	0.10 (0.18)	[–0.24, 0.45]	0.559
Living Situation	0.18 (0.18)	[–0.18, 0.53]	0.326	0.47 (0.21)	[0.06, 0.89]	0.026
Increased Care	–0.22 (0.25)	[–0.70, 0.26]	0.371	0.21 (0.25)	[–0.35, 0.78]	0.459
Supervisory Function	–0.45 (0.17)	[–0.78, –0.13]	0.006	–0.35 (0.29)	[–0.73, 0.03]	0.073
Days in Lockdown	–0.01 (0.01)	[–0.03, 0.02]	0.699	–0.04 (0.19)	[–0.07, –0.00]	0.032
Normal Job Hours	0.02 (0.01)	[0.01, 0.03]	0.001	0.01 (0.01)	[–0.00, 0.03]	0.130
Work % Left	0.00 (0.00)	[–0.00, 0.00]	0.899	0.01 (0.00)	[0.00, 0.01]	0.014
Model	*N* = 408; *R*^2^ = 0.08, *F* (8, 399) = 4.38, *p* < 0.001				*N* = 408; *R*^2^ = 0.06, *F* (8, 399) = 3.35, *p* < 0.001	

**Table 5:** Multiple regression analysis of work percentage left on identification.

	Identification with Organization			Identification with Colleagues		
	*B (SE B)*	95% CI	*p*	*B (SE B)*	95% CI	*p*
Age	0.02 (0.01)	[0.01, 0.03]	0.002	0.02 (0.01)	[0.01, 0.03]	0.000
Gender	0.22 (0.17)	[–0.10, 0.55]	0.178	0.32 (0.15)	[0.02, 0.61]	0.037
Living Situation	0.19 (0.07)	[–0.21, 0.58]	0.354	0.20 (0.18)	[–0.16, 0.56]	0.269
Increased Care	–0.17 (0.20)	[–0.70, 0.37]	0.537	–0.10 (0.25)	[–0.58, 0.39]	0.703
Supervisory Function	–0.37 (0.27)	[–0.73, –0.01]	0.043	–0.47 (0.17)	[–0.80, –0.14]	0.005
Days in Lockdown	–0.02 (0.18)	[–0.05, 0.01]	0.151	–0.01 (0.02)	[–0.04, 0.02]	0.491
Normal Job Hours	0.01 (0.01)	[–0.01, 0.02]	0.197	0.01 (0.01)	[0.00, 0.02]	0.055
Work % Left	0.01 (0.00)	[0.00, 0.01]	0.015	0.00 (0.00	[–0.00, 0.01]	0.128
Model	*N* = 408; *R*^2^ = 0.08, *F* (8, 399) = 4.44, *p* < 0.001			*N* = 408; *R*^2^ = 0.10, *F* (8, 399) = 5.58, *p* < 0.001		

**Table 6:** Multiple regression analysis of work percentage left on job insecurity.

	Job Insecurity		
	*B (SE B)*	95% CI	*p*
Age	–0.00 (0.00)	[–0.02, 0.01]	0.558
Gender	0.25 (0.21)	[–0.17, 0.67]	0.249
Living Situation	–0.36 (0.26)	[–0.87, 0.15]	0.166
Increased Care	–0.11 (0.35)	[–0.80, 0.58]	0.757
Supervisory Function	0.19 (0.24)	[–0.28, 0.65]	0.425
Days in Lockdown	0.07 (0.02)	[0.03, 0.11]	0.001
Normal Job Hours	0.01 (0.01)	[–0.01, 0.03]	0.199
Work % Left	–0.02 (0.00)	[–0.02, –0.01]	0.000
Model	*N* = 408; *R*^2^ = 0.13, *F* (8, 399) = 7.20, *p* < 0.001		

**Table 7:** Multiple regressions of work perceptions on online OCB-I and online OCB-O.

	Online OCB-I			Online OCB-O		
	*B (SE B)*	95% CI	*p*	*B (SE B)*	95% CI	*p*
Age	–0.00 (0.01)	[–0.01, 0.01]	0.433	–0.02 (0.01)	[–0.03, –0.01]	0.002
Gender	0.03 (0.16)	[–0.28, 0.34]	0.849	0.28 (0.18)	[–0.07, 0.63]	0.114
Living Situation	–0.15 (0.19)	[–0.51, 0.22]	0.426	0.03 (0.21)	[–0.38, 0.45]	0.879
Increased Care	–0.28 (0.24)	[–0.76, 0.20]	0.257	0.20 (0.28)	[–0.34, 0.74]	0.458
Supervisory Function	–0.55 (0.17)	[–0.88, –0.21]	0.001	–0.23 (0.19)	[–0.61, 0.15]	0.234
Days in Lockdown	–0.02 (0.02)	[–0.05, 0.01]	0.235	0.03 (0.02)	[–0.01, 0.06]	0.127
Normal Job Hours	0.01 (0.01)	[–0.00, 0.03]	0.069	0.01 (0.01)	[–0.00, 0.03]	0.195
Work % Left	0.00 (0.00)	[–0.00, 0.01]	0.107	–0.00 (0.00)	[–0.01, 0.00]	0.546
Normal Telework %	0.01 (0.00)	[0.01, 0.02]	0.000	0.00 (0.00)	[–0.00, 0.01]	0.426
Increase Telework %	0.01 (0.00)	[0.01, 0.02]	0.000	0.00 (0.00)	[–0.01, 0.00]	0.838
Positive Meaning	0.15 (0.07)	[0.01, 0.29]	0.033	0.12 (0.08)	[–0.04, 0.28]	0.139
Greater-Good	–0.06 (0.05)	[–0.16, 0.04]	0.221	0.01 (0.06)	[–0.11, 0.12]	0.914
Identification (O)	–0.05 (0.08)	[–0.21, 0.11]	0.517	0.18 (0.09)	[0.00, 0.36]	0.048
Identification (C)	0.28 (0.08)	[0.13, 0.43]	0.000	0.11 (0.09)	[–0.07, 0.28]	0.223
Job Insecurity	0.07 (0.04)	[–0.00, 0.14]	0.054	0.12 (0.04)	[0.04, 0.20]	0.004
Model	*N* = 318; *R*^2^ = 0.27, *F* (15, 302) = 7.48, *p* < 0.001			*N* = 318; *R*^2^ = 0.18, *F* (15, 302) = 4.37, *p* < 0.001		

**Table 8:** Multiple regression analysis of work perceptions on cyberostracism and cyberincivility.

	Cyberostracism			Cyberincivility		
	*B (SE B)*	95% CI	*p*	*B (SE B)*	95% CI	*p*
Age	–0.01 (0.00)	[–0.02, –0.01]	0.000	–0.00 (0.00)	[–0.01, 0.00]	0.546
Gender	–0.07 (0.09)	[–0.25, 0.12]	0.483	–0.11 (0.06)	[–0.23, 0.02]	0.094
Living Situation	0.25 (0.11)	[0.03, 0.46]	0.027	0.04 (0.07)	[–0.10, 0.19]	0.571
Increased Care	0.02 (0.14)	[–0.27, 0.30]	0.918	0.10 (0.10)	[–0.09, 0.29]	0.288
Supervisory Function	–0.09 (0.10)	[–0.29, 0.11]	0.381	–0.20 (0.07)	[–0.33, 0.07]	0.003
Days in Lockdown	–0.00 (0.01)	[–0.02, 0.01]	0.656	–0.01 (0.01)	[–0.02, 0.01]	0.273
Normal Job Hours	0.01 (0.00)	[–0.00, 0.02]	0.072	0.00 (0.00)	[–0.00, 0.01]	0.131
Work % Left	0.00 (0.00)	[–0.00, 0.00]	0.857	–0.00 (0.00)	[–0.00, 0.00]	0.439
Normal Telework %	0.00 (0.00)	[0.00, 0.01]	0.026	0.00 (0.00)	[–0.00, 0.00]	0.296
Increase Telework %	0.00 (0.00)	[–0.00, 0.00]	0.997	0.00 (0.00)	[–0.00, 0.00]	0.724
Positive Meaning	–0.01 (0.04)	[–0.10, 0.07]	0.756	–0.02 (0.03)	[–0.08, 0.03]	0.413
Greater-Good	–0.02 (0.03)	[–0.08, 0.04]	0.602	0.00 (0.02)	[–0.04, 0.04]	0.852
Identification (O)	–0.07 (0.05)	[–0.16, 0.02]	0.142	–0.06 (0.03)	[–0.12, 0.01]	0.070
Identification (C)	0.01 (0.05)	[–0.09, 0.10]	0.914	0.01 (0.03)	[–0.05, 0.08]	0.664
Job Insecurity	0.05 (0.02)	[0.01, 0.10]	0.012	0.03 (0.01)	[0.00, 0.06]	0.027
	*N* = 318; *R^2^* = 0.13, *F* (15, 302) = 3.01, *p* < 0.001			*N* = 318; *R^2^* = 0.11, *F* (15, 302) = 2.60, *p* = 0.001		

## Data Availability

The data that support the findings of this study are available from the corresponding author upon reasonable request.

## Appendix A. Exploratory Factor Analysis of Online Organizational Behaviors

Because the items were non-normally distributed, we opted for a principal axis factor extraction and a promax rotation with Kaiser normalization in line with recommendations e.g., [81]. The Kaiser-Meyer-Olkin (KMO) measure of sampling adequacy (.82) indicated that the data were appropriate for analysis. The scree test suggested that four factors should be retained. All four factors had an eigenvalue greater than 1 and together accounted for 63.47% of the variance. With the exception of one item intended to assess OCB-I and one item intended to assess OCB-O, all items clearly loaded on the intended factor. Accordingly, after removing these two items, scales were constructed to measure online OCB-I (M = 4.64, SD = 1.40, α = 0.80), online OCB-O (M = 3.60, SD = 1.49, α = 0.73), cyberincivility (M = 1.25, SD = 0.51, α = 0.80), and cyberostracism (M = 1.53, SD = 0.76, α = 0.75).

**Table A1:** Exploratory factor analysis with promax rotation of online organizational behaviors (pattern matrix).

	Component			
*How often do you …*	1	2	3	4
Help direct colleagues via email, chat, videoconferencing or social media when they have a work-related problem?		0.82		
Make suggestions to direct colleagues via email, chat, videoconferencing or social media to make the performance of their job easier?		0.93		
Support your direct colleagues via email, chat, videoconferencing or social media when they feel uncomfortable or have a bad day?		0.54		
Respond positively to messages of direct colleagues on social media (for example, by “liking” them)? *			0.76	
Speak positively about your organization via email, chat, videoconferencing or social media?			0.71	
Make suggestions for improvements within your organization via email, chat, videoconferencing or social media?*		0.42	0.32	
Defend your organization via email, chat, videoconferencing or social media?			0.51	
Respond positively to messages of your organization on social media (for example, by “liking” them)			0.76	
Deliberately not forward an email to someone at work, although you know this is important for him or her?				0.46
On purpose omit information in an email to someone at work, although you know this information is important to him or her?				0.69
Deliberately wait a long time when responding to an email that is directed to you personally from someone at work?				0.78
Completely ignore an email that is directed to you personally from someone at work?				0.74
Send an impolite email to someone at work?	0.69			
Make fun of a colleague in an email?	0.85			
Send an angry email to someone at work?	0.71			
Use CAPS in an email to someone at work to “shout’”	0.67			
Eigenvalue	4.20	3.15	1.44	1.37
Cumulative % of variance explained	26.24	45.92	54.90	63.47

*Note:* Only factor loadings > 0.20 are depicted; cross-loadings in italics; * item deleted from scale.
